# Calcified Intervertebral Disc Herniation in a Child

**DOI:** 10.5334/jbsr.3228

**Published:** 2023-08-09

**Authors:** Aseel Al-Musaedi, Filip M. Vanhoenacker, Anton Veyt

**Affiliations:** 1 Department of radiology, AZ Sint-Maarten, Mechelen, BE; 2 AZ Sint-Maarten and University (Hospital) Antwerp/Ghent, BE; 3 Department of Neurosurgery, AZ Monica, Deurne, BE

**Keywords:** Intervertebral disc calcification, Paediatric, Cervical spine, CT, MRI

## Abstract

**Teaching Point:** Calcified intervertebral disc herniation in children is rare, and spontaneous resolution is the rule.

## Case History

A 9-year-old boy presented to the emergency department with neck pain and stiffness for two weeks. The complaints started with fever, decreased appetite and vomiting. The pain increased gradually over the next few days and did initially not respond to analgesics. Clinical examination revealed limited range of motion (ROM). Neurological examination was normal. Laboratory examination was unremarkable except for mild leucocytosis (13.8 × 10^3^/µL).

**Figure 1:**
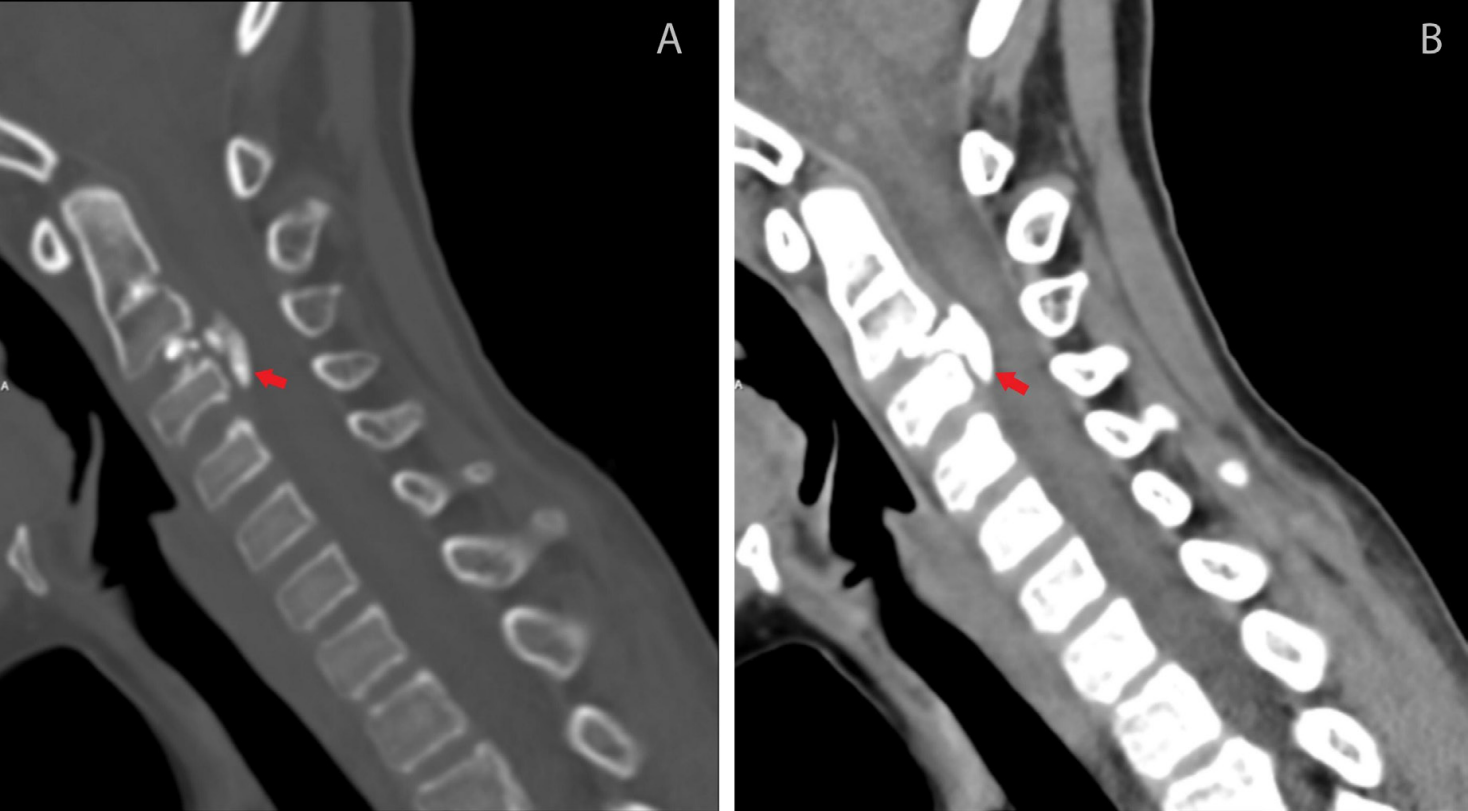


**Figure 2:**
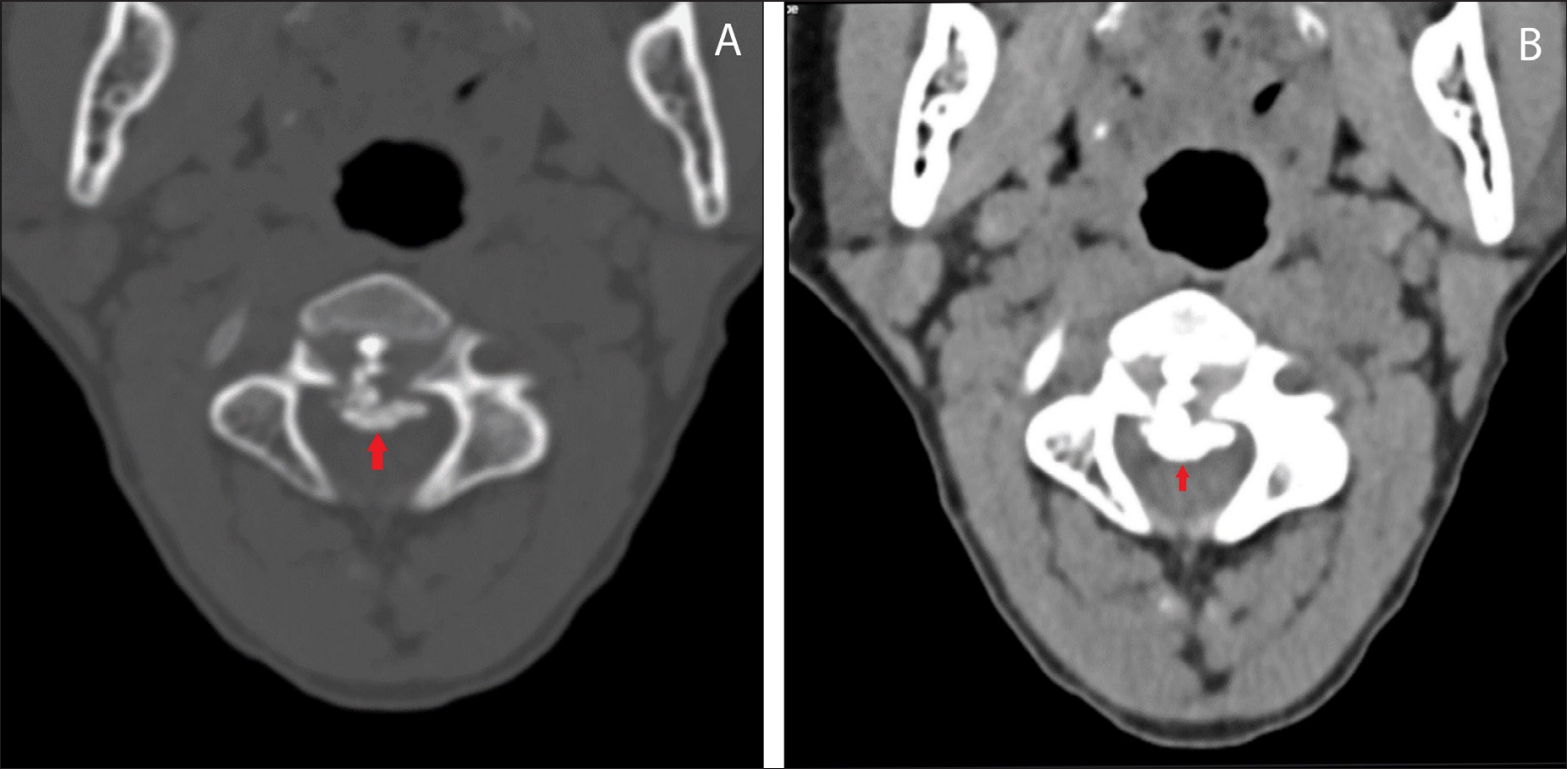


On computed tomography (CT), there was calcification of the intervertebral disc (IVD) of C2-C3, with a significant herniation to the anterior epidural space with inferior migration underneath the posterior longitudinal ligament (Figure 1A, bone window; Figure 1B, soft tissue window, red arrows). Axial CT revealed mass effect on the spinal cord (Figure 2A, bone window; Figure 2B, soft tissue window, red arrows).

**Figure 3:**
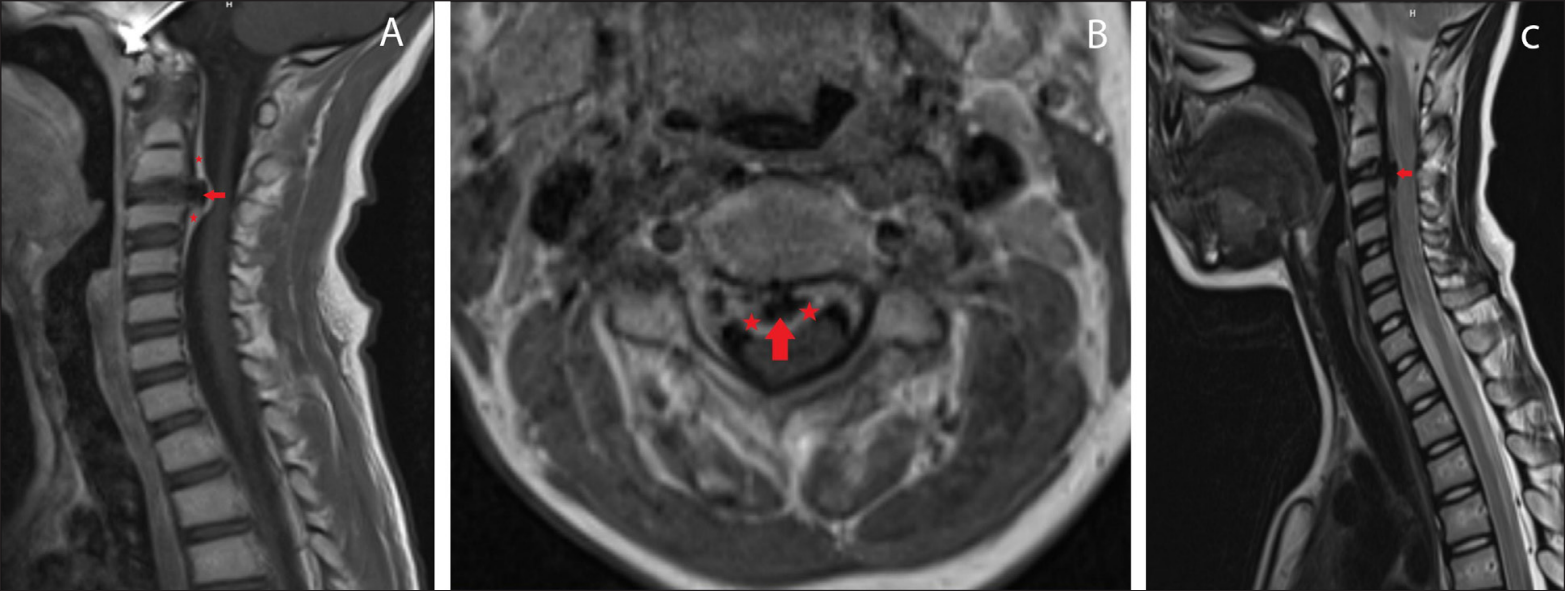


Subsequent magnetic resonance imaging (MRI) showed peripheral epidural enhancement surrounding the calcified disc with a tail-like extension superiorly and inferiorly (Figure 3A–B, sagittal and axial T1-weighted images [WI] after gadolinium contrast respectively, red arrow: herniation, asterisks: enhancement). Spinal cord compression is best appreciated on the axial image. Sagittal T2-WI revealed low signal intensity of the calcified disc herniation (Figure 3C, red arrow). The patient was treated conservatively with analgesics and a cervical collar. There was a significant improvement with gradual resolution of pain. Follow-up MRI four months later demonstrated nearly complete resolution of the extruded calcified disc.

## Comments

Although intervertebral disc (IVD) calcification is a common finding at imaging in adults, it is rare in children.

It can be asymptomatic or symptomatic. Seventy per cent of the symptomatic cases occurs in the cervical spine [1]. The mean age of paediatric patients is seven years with a male predominance [1]. Although (micro)traumatic, infectious, metabolic and inflammatory mechanisms are thought to contribute to the pathogenesis of calcification of IVD in children, its etiology is still a matter of debate.

Patients present with insidious cervical pain and stiffness, sometimes with mild inflammatory signs. Radiographs or CT reveal a calcified nucleus pulposus, with or without disc herniation On MRI imaging, the calcified disc is hypointense on both T1 and T2-WI. Sometimes adjacent edema and enhancement due to inflammation can be seen, like in our case.

Follow-up imaging shows complete resolution in most cases, with resorption of the calcifications. Residual changes that have been observed radiographically in some patients include vertebral body flattening, residual disk space calcification, and osteophytes.

The prognosis of this condition is favourable, and therefore most cases can be treated conservatively without surgical intervention.

Very rarely, symptomatic compression of the neural element requires surgery.

The radiologist should be aware of this paediatric condition because it has a favourable prognosis. Invasive diagnostic or therapeutic intervention should be avoided.
